# *Mos1* Element-Mediated CRISPR Integration of Transgenes in *Caenorhabditis elegans*

**DOI:** 10.1534/g3.119.400399

**Published:** 2019-06-11

**Authors:** Nicholas S. Philip, Fernando Escobedo, Laura L. Bahr, Brandon J. Berry, Andrew P. Wojtovich

**Affiliations:** * Department of Anesthesiology and Perioperative Medicine, and; † Department of Pharmacology & Physiology, University of Rochester Medical Center, Rochester NY, 14642

**Keywords:** *C. elegans*, CRISPR/Cas9, *Mos1*-mediated single copy integration, homology-directed repair

## Abstract

The introduction of exogenous genes in single-copy at precise genomic locations is a powerful tool that has been widely used in the model organism *Caenorhabditis elegans*. Here, we have streamlined the process by creating a rapid, cloning-free method of single-copy transgene insertion we call *Mos1* element-mediated CRISPR integration (mmCRISPi). The protocol combines the impact of *Mos1* mediated single-copy gene insertion (mosSCI) with the ease of CRISPR/Cas9 mediated gene editing, allowing *in vivo* construction of transgenes from linear DNA fragments integrated at defined loci in the *C. elegans* genome. This approach was validated by defining its efficiency at different integration sites in the genome and by testing transgene insert size. The mmCRISPi method benefits from *in vivo* recombination of overlapping PCR fragments, allowing researchers to mix-and-match between promoters, protein-coding sequences, and 3′ untranslated regions, all inserted in a single step at a defined *Mos1* loci.

Genetic approaches rely on complementation of mutant phenotypes, often times using a transgene that has been epitope-tagged for visualization, or that is expressed in individual tissues to allow its site of action to be determined. However, these approaches can be confounded by overexpression artifacts. For example, canonical microinjection techniques used to complement *C. elegans* mutants result in strains that contain an extrachromosomal array, consisting of numerous of copies of the recombinant transgene in tandem repeat arrays. In addition, extrachromosomal arrays are lost during cell division at a variable rate that differs between individual transgenes, resulting in genetic mosaicism. This can be circumvented by integrating the transgene into the genome, which also limits genetic instability and stabilizes expression levels. There are various processes for integrating arrays, with methods that involve inducing a chromosomal break followed by insertion and repair, including using CRISPR/Cas9 (Yoshina *et al.* 2016). However, mechanisms such as UV-mediated integration can be imprecise and can result in the integration of multiple transgene copies or the interruption of native genes (Kage-Nakadai *et al.* 2014). *Mos1*-mediated Single Copy Insertion (mosSCI) overcomes the limitations of UV-mediated integration, and expresses genes of interest at close to endogenous levels at defined loci (Frøkjær-Jensen *et al.* 2012; Frøkjær-Jensen *et al.* 2008). A large cohort of *C. elegans* strains has been developed containing *Drosophila mauritiana Mos1* transposon inserted at defined sites in the *C. elegans* genome. mosSCI uses the *Mos1* transposase to excise the *Mos1* transposon, resulting in a double strand DNA break that can be repaired using exogenous templates that contain a gene of interest. This approach is effective, but it is also time intensive and requires extensive cloning (Frøkjær-Jensen *et al.* 2012). Hence, we set out to improve single copy transgene insertion methods by combining elements of mosSCI with the advances in CRISPR/Cas9 genome editing.

The CRISPR/Cas9 system allows for precise genome insertions or deletions. A twenty base CRISPR RNA (crRNA) directs Cas9 to make a double strand break at the target site, which can be repaired through homology-directed repair (HDR). Techniques to modify the *C. elegans* genome are becoming increasingly efficient and no longer require cloning (Paix *et al.* 2015; Arribere *et al.* 2014). However, these methods require users to select a genomic locus to direct Cas9 that is transcriptionally active and amenable to exogenous gene insertion, which may be difficult to determine. Here we combine the advances of mosSCI and CRISPR/Cas9 techniques, and describe the technique we call *Mos1* transposon-mediated CRISPR integration (mmCRISPi). We use the cloning-free approach described for HDR with CRISPR/Cas9 ribonucleoprotein complexes and target Cas9 (Paix *et al.* 2015) to the *Mos1* element in available mosSCI *C. elegans* strains (Frøkjær-Jensen *et al.* 2008; Frøkjær-Jensen *et al.* 2012). A gene can be constructed from multiple linear PCR fragments through a process termed recombineering (Kemp *et al.* 2007; Paix *et al.* 2016). Tested transgene insertions showed similar expression levels across strains, suggesting predominantly single-copy insertions. Collectively, this approach is a rapid and efficient method to integrate transgenes at defined euchromatic loci.

## Materials and Methods

### C. elegans strains

*C. elegans* strains were grown at 20° on nematode growth media (NGM) and OP50 *E*. *coli* bacteria as a source of food using standard laboratory techniques (Brenner 1974). mosSCI insertion strains (EG6699, EG6701 and EG6703) (Frøkjær-Jensen *et al.* 2012) were obtained from the *Caenorhabditis Genetics Center* (CGC, University of Minnesota, Minneapolis, MN, USA). All mosSCI insertion strains were outcrossed to N2 (Table 1) and non-unc and non-fluorescent worms were selected. The resulting strains were homozygous for the *Mos1* transposon. Strains and plasmids are available upon request and from the *C. elegans* Genetics Center. The authors affirm that all data necessary for confirming the conclusions of the article are present within the article, figures, and tables.

**Table 1 C.** *elegans* strains. Strains generated in this study were crossed to N2 removed the *unc-119(ed3)* phenotype. Strains were provided by the *C. elegans* Genetics Center (CGC)

Strain	Genotype	Source
EG6699	*ttTi5605 II*; *unc-119(ed3) III*; *oxEx1578.*	CGC
EG6701	*ttTi4348 I*; *unc-119(ed3) III*; *oxEx1580*	CGC
EG6703	*unc-119(ed3) III*; *cxTi10816 IV*; *oxEx1582.*	CGC
APW65	*ttTi5605 II*	This study
APW109	*ttTi4348 I*	This study
APW156	*cxTi10816 IV*	This study

### CRISPR/Cas9 editing

CRISPR/Cas9 editing in *C. elegans* was followed as described by *Paix et al* using the *dpy-10* co-CRISPR method (Paix *et al.* 2015). Briefly, Cas9 was purified from BL21(DE3)pLysS cells (NEB) expressing pHO4d-Cas9 (gift from Michael Nonet; Addgene plasmid # 67881; http://n2t.net/addgene:67881; RRID:Addgene_67881)(Fu *et al.* 2014) using Ni-NTA agarose beads (Trewin *et al.* 2019). The *Mos1* transposon was sequenced and three crRNA were selected using a guide design tool (crispr.mit.edu). crRNA (Table 2) and *trans*-activating RNA (tracrRNA) were purchased from Dharmacon.

**Table 2:** crRNA for CRISPR-Homology Directed Repair editing. crRNAs were selected using the guide design tool, crispr.mit.edu

Gene Target	crRNA target sequence
*crRNA1*	*CTATGGTGGTTCGACAGTCA*
*crRNA2*	*GTCCGCGTTTGCTCTTTATT*
*crRNA3*	*CCCATCTCTCGGGCAATTTG*

Repair template primers and *dpy-10* single-stranded oligonucleotides (ssODN) were purchased from Integrated DNA technologies (Table 3). Repair templates were PCR amplified and purified using a Qiagen minelute column (Paix *et al.* 2015). Each end of the repair template (*i.e.*, PCR amplicon) contained 35 bp of homology to either the Cas9 cut site or a subsequent fragment. Injection mixes contained 4 µg/µL tracrRNA, 0.8 µg/µL target crRNA, 0.1 µg/µL *dpy-10* crRNA, 50 ng/µL *dpy-10* ssODN, 7.5 mM HEPES pH 7.4, 25 mM KCl, 2.5 µg/µL Cas9 and repair PCR amplicon(s). Each repair PCR amplicon within a set was present at equal molar concentrations (0.147-0.443 pmol/uL; see legend for details).

**Table 3:** Amplicon repair template primers. Forward and reverse primer sequences used to make amplicons noted in each figure legend. Note some primers were used to generate more than one amplicon

Name	Primer Sets	Template	Product
Figure 2 Amplicon 1	*F: cataaaactttgaccttgtgaagtgtcaaccttgaagtgattatagtctctgttttcg*	pCFJ70	*P_myo-3_*
(2.1)	*R:ctcaccattaagcctgcttttttgtacaaacttgtcaattctagatggatctagtggtcg*		
Figure 2 Amplicon 2	*F: acaagtttgtacaaaaaagcaggcttaatggtgagcaagggcgaggagctg*	T5V - pTurquoise_5aa_Venus	*Venus*
(2.2)	*R: gtacaagaaagctgggtactagatccggtggatcccgg*		
Figure 2 Amplicon 3	*F: ccgggatccaccggatctagtacccagctttcttgtac*	pCFJ90	*unc-54 UTR*
(2.3)	*R:gctcaattcgcgccaaactatggtggttcgacagaaacagttatgtttggtatattg*		
Figure 2 Amplicon 4	*F:tcagtgcagtcaacatgtcgagtttcgtgccgaatagtgattatagtctctgttttcgttaattttg*	pCFJ70	*P_myo-3_*
(2.4)	*R:ctcaccattaagcctgcttttttgtacaaacttgtcaattctagatggatctagtggtcg*		
Figure 2 Amplicon 5	*F: ccgggatccaccggatctagtacccagctttcttgtac*	pCFJ90	*unc-54 UTR*
(2.5)	*R: aaacagaaaattaatactgtccgcgtttgctctttaacagttatgtttggtatattg*		
Figure 2 Amplicon 6	*F:aaagacgatgagttctactggcgtggaatccacaaagtgattatagtctctgttttcg*	pCFJ70	*P_myo-3_*
(2.6)	*R:ctcaccattaagcctgcttttttgtacaaacttgtcaattctagatggatctagtggtcg*		
Figure 2 Amplicon 7	*F: ccgggatccaccggatctagtacccagctttcttgtac*	pCFJ90	*unc-54 UTR*
(2.7)	*R: cgctagctacacatttttcccatctctcgggcaataacagttatgtttggtatattg*		
Figure 3 Amplicon 1	*F:tcagtgcagtcaacatgtcgagtttcgtgccgaatcattttatatctgagtagtatcctttgc*	pCFJ90	*P_myo-2_*
(3.1)	*R: ttactcattaagcctgcttttttgtacaaacttgt*		
Figure 3 Amplicon 2	*F:acaagtttgtacaaaaaagcaggcttaatgagtaaaggagaagaac*	pCZGY1614	*GFP*
(3.2)	*R:aaacagaaaattaatactgtccgcgtttgctctttttatttgtatagttcatccatg*		
Figure 3 Amplicon 3	*F:acaagtttgtacaaaaaagcaggcttaatgagtaaaggagaagaac*	pCZGY1614	*GFP*
(3.3)	*R:tacaagaaagctgggtattatttgtatagttcatccatg*		
Figure 3 Amplicon 4	*F: Gatgaactatacaaataatacccagctttcttgtac*	pCFJ90	*unc-54 UTR*
(3.4)	*R: aaacagaaaattaatactgtccgcgtttgctctttaacagttatgtttggtatattg*		
Figure 3 Amplicon 5	*F:acaagtttgtacaaaaaagcaggcttaatgagtaaaggagaagaac*	pCZGY1614	*GFP*
(3.5)	*R:gagaccatcgatgctcctgaggctcccgatgctcctttgtatagttcatccatgcc*		
Figure 3 Amplicon 6	*F:ggagcatcgggagcctcaggagcatcgatggtctcaaagggtgaagaag*	pCFJ90	*Cherry*
(3.6)	*R:ccgatgcggagctcagatatcacccactttgtaca*		
Figure 3 Amplicon 7	*F: tgtacaaagtgggtgatatctgagctccgcatcgg*	pCFJ90	*unc-54 UTR*
(3.7)	*R: aaacagaaaattaatactgtccgcgtttgctctttaacagttatgtttggtatattg*		
Figure 4 Amplicon 1	*F:tcagtgcagtcaacatgtcgagtttcgtgccgaatgacgacgacgacctcgacggcaac*	pSEP45	*P_rab-3_*
(4.1)	*R:gccatttttaagcctgcttttttgtacaaacttgtctgaaaatagggctactgtag*		
Figure 4 Amplicon 2	*F:acaagtttgtacaaaaaagcaggcttaaaaatggctgcgttcttgctgagac*	pBB34	*Sdhc*::*MAC*
(4.2)	*R:ggatcctcctcctccagatcctcctccacctcgggcgccgtcgtcctcgccgatc*		
Figure 4 Amplicon 3	*F:cccgaggtggaggaggatctggaggaggaggatccatggtttccgagttgatcaagg*	pKT133	*mKate*
(4.3)	*R:ttaacgatgtccgagcttggatgggagatcacaatatc*		
Figure 4 Amplicon 4	*F:attgtgatctcccatccaagctcggacatcgttaagtccaattactcttcaac*	pCFJ90	*unc-54 UTR*
(4.4)	*R: aaacagaaaattaatactgtccgcgtttgctctttaaggtattttgtgtgcgg*		
Figure 4 Amplicon 5	*F:tcagtgcagtcaacatgtcgagtttcgtgccgaatagcacagaactgcattaag*	pELA10	*P_vha-6_*
(4.5)	*R:gccatttttaagcctgcttttttgtacaaacttgtatttttatgggttttggtag*		
Figure 4 Amplicon 6	*F: tcagtgcagtcaacatgtcgagtttcgtgccgaatagtgattatagtctctgttttc*	pAYW7	*P_myo-3_*
(4.6)	*R: gccatttttaagcctgcttttttgtacaaacttgtcaattctagatggatctagtg*		

Each mix was injected into the gonad of 20-30 day 1 adults containing a *Mos1* element. Injected adults were transferred to individual plates and incubated at 20°. After about 5 days, plates were sorted for a successful injection as determined by a large number of *dpy-10* F1 progeny. The F1 progeny from these plates were scored for *dpy-10* edits (*dpy*, observable dumpy/roller phenotype) and expected promoter-dependent fluorescent pattern (Paix *et al.* 2016; Paix *et al.* 2015). Fluorescent and *dpy* F1 worms were singled. About 3 days later, F2 progeny were assessed for fluorescent transmission. F1 worms with fluorescent progeny were then PCR genotyped to confirm integration. To account for differences in Cas9 editing and *in vivo* array formation between experiments, integration efficiency was calculated as the percent of fluorescent *dpy-10*-edited F1 progeny that had a PCR confirmed integration of the engineered fluorescent transgene divided by the total fluorescent *dpy-10*-edited F1 progeny (Paix *et al.* 2016).

### Fluorescence microscopy

Whole animal images were acquired on an Olympus MVX10 Fluorescence MacroZoom dissecting microscope, using a 540-580 nm excitation filter and 590-670 nm emission filter. Mitochondrial images were taken on a FV1000 Olympus laser scanning confocal microscope using a 100x oil objective (Olympus, N.A. 1.40). Diode laser illumination was 561 nm for red fluorescent transgene and 488 nm for fluorescence of MitoTracker Green FM. Animals were stained with 12 μM MitoTracker Green FM for 20 hr where indicated. MitoTracker stain was dissolved in DMSO, diluted in M9 media (22 mM KH_2_PO_4_, 42 mM Na_2_HPO_4_, 86 mM NaCl, 1 mM MgSO_4_, pH 7) and added to OP50 food on culture plates (DMSO <0.02% final concentration) and allowed to dry (Dingley *et al.* 2010). Profile plots of pixel intensity were generated using ImageJ software.

## Results

### Mos1 crRNA efficacy using recombineering with linear PCR fragments

**Figure 1:**
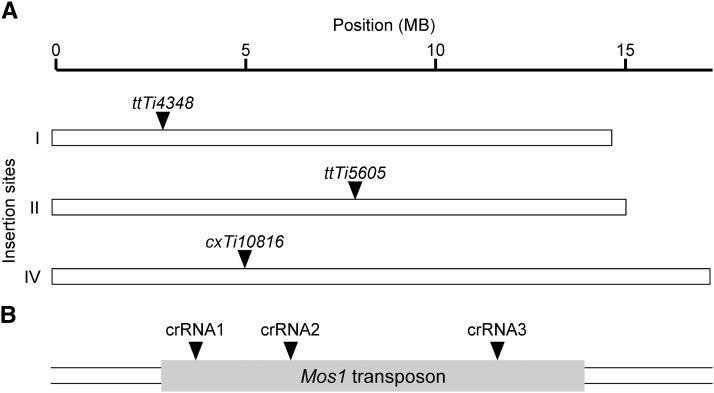
*Mos1*-mediated CRISPR integration (mmCRISPi) schematic. A) The mosSCI system has insertion strains containing a *Mos1* transposon at defined loci. We use strains harboring transposon insertion alleles *ttTi4348*, *ttTi5605*, and *cxT10816* with a *Mos1* transposon on chromosomes I, II and IV, respectively. B) We targeted Cas9 to the *Mos1* transposon using crRNA1, crRNA2, or crRNA3.

**Figure 2:**
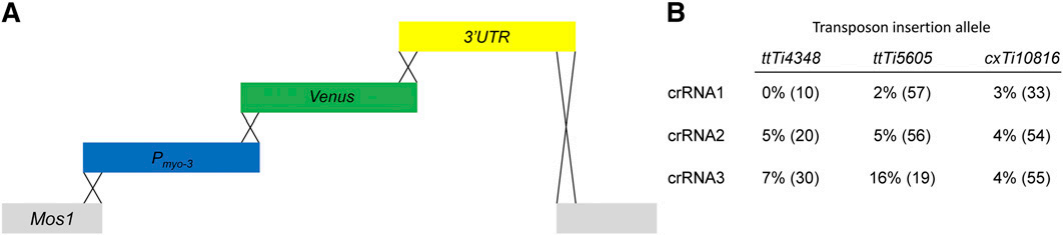
*Mos1* crRNA efficacy using recombineering with linear PCR fragments. A) Schematic (not to scale). A Cas9-mediated double strand break in the *Mos1* transposon sequence (gray) was induced using crRNA1, crRNA2, or crRNA3. The break was repaired with three PCR amplicons to drive Venus expression in the body wall muscles. Crossed lines indicate homologous DNA sequences, which overlap by 35 bp. *P_myo-3_* (blue, 2573 bp), *Venus* (Green, 810 bp) and *unc-54 3′ UTR* (yellow, 853 bp). B) The number of successfully injected P0 broods scored for crRNA1 was 3, 13, and 7 for *ttTi4348*, *ttTi5605* and *cxTi10816*, respectively; crRNA2, 9, 13, 13; crRNA3, 5, 3, 10. The resulting *dpy-10* progeny were screened for yellow fluorescent body wall muscles. Data are the percent integrations among the fluorescent *dpy-10* edits. The number of fluorescent *dpy-10* worms screened is in parentheses. The total F1 *dpy-10* edits (non-fluorescent plus fluorescent) screened for crRNA1 was 30, 123, and 85 for *ttTi4348*, *ttTi5605* and *cxTi10816*, respectively; crRNA2, 177, 199, 264; crRNA3, 74, 38, 141. PCR amplicons were injected at 0.147 - 0.210 pmol/µl in mixes containing crRNA1, 0.202 - 0.217 pmol/µl in mixes containing crRNA2, and 0.147 - 0.215 pmol/µl in mixes containing crRNA3. All PCR amplicons are listed in Table 3. PCR amplicons for crRNA1 are 2.1 (*P_myo-3_*), 2.2 (*Venus*), and 2.3 (*3′ UTR*) for crRNA1; 2.4 (*P_myo-3_*), 2.2 (*Venus*), and 2.5 (*3′ UTR*) for crRNA2; 2.6 (*P_myo-3_*), 2.2 (*Venus*), 2.7 (*3′ UTR*) for crRNA3.

A versatile transgene expression system would be easily adapted to various integration sites throughout the genome. We reasoned that a crRNA targeted within the *Mos1* transposon (NCBI accession number X78906) would allow a user to integrate a transgene on the chromosome of choice by simply switching the insertion strain (Figure 1). This would facilitate a streamlined approach that allows multiple *Mos1* insertions to be created using the same reagents. Since target choice strongly impacts CRISPR/Cas9 effectiveness (Farboud and Meyer 2015; Doench *et al.* 2014), three separate crRNA sequences (crRNA1, crRNA2 and crRNA3, see Table 2) were chosen following established criteria (Gagnon *et al.* 2014; Hsu *et al.* 2013). The individual crRNAs were compared by measuring the CRISPR/Cas9 mediated integration of a fluorescent reporter transgene following established *C. elegans* methodology with purified Cas9 protein, *in vitro* synthesized ribonucleoprotein complexes, and a *dpy-10* co-CRISPR selection (Arribere *et al.* 2014; Paix *et al.* 2015; Cho *et al.* 2013). The reporter consisted of three overlapping PCR fragments: the *myo-3* promoter to direct body wall muscle expression, the coding sequence of Venus, a yellow fluorescent protein, for visualization, and the *unc-54* 3′ untranslated region (UTR) to stabilize the mRNA, each with ∼35 bases of homology to facilitate *in vivo* recombination with the adjacent PCR fragment or genomic *Mos1* site (Figure 2A). In order to distinguish homologous recombinants from extrachromosomal arrays (Kemp *et al.* 2007), we followed a validation scheme that took advantage of the *dpy-10* co-CRISPR strategy (Paix *et al.* 2016). F2 progeny from *dpy-10* co-CRISPR founders were scored for Mendelian inheritance of the fluorescent reporter, and the F1 parent was PCR genotyped at the *Mos1* locus to confirm integration (Figure 2B). We calculated the percent integration events among the fluorescent *dpy-10* worms. This scoring scheme accounts for both successful CRISPR/Cas9 edits and *in vivo* formation of the transgene (*i.e.*, recombineering). Comparing the three crRNAs tested, we found both crRNA 2 and 3 to be relatively efficient compared to crRNA1 (Figure 2B). In addition, we found that the transposon insertion allele on chromosome II (*ttTi5605*) yielded more integration events than the other loci tested. However, the *ttTi56505* allele is in close proximity to *dpy-10*, which could make outcrossing to remove the *dpy-10* co-CRISPR point mutant difficult. Therefore, we examined the chromosome I integration site (*ttTi4348* allele, see Figure 1) in more detail.

### DNA recombineering with altered size and fragment number

**Figure 3:**
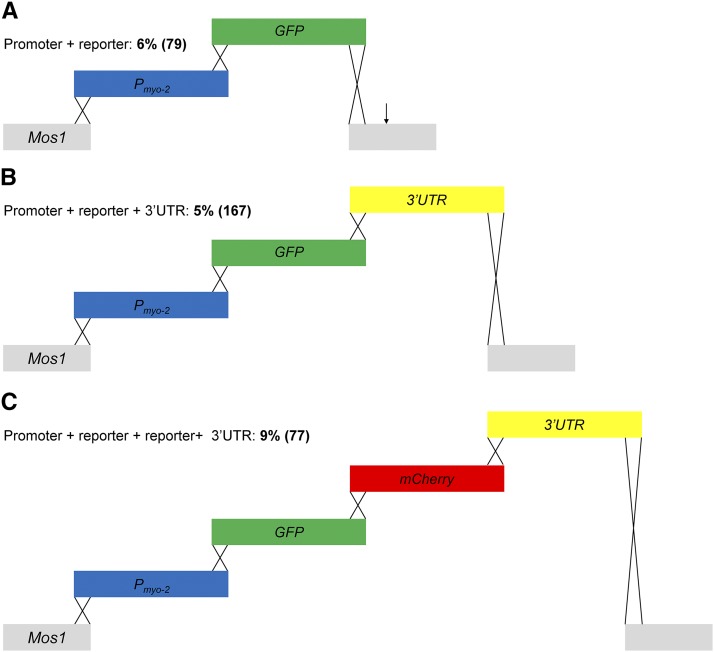
DNA recombineering with altered size and fragment number. Schematics (not to scale) show the double strand break in the *Mos1 element* (gray) using crRNA2 in the *ttTi4348* transposon allele. A) Promoter + reporter: Two PCR amplicons encoding the *myo-2* promoter (blue) or *GFP* (green) were injected into the *C. elegans* gonad. The homology regions overlap by 35 bases and are indicated by crossed lines. A potential polyadenylation signal (PAS) intrinsic to the *Mos1 element* (arrow) was downstream of the crRNA2 cut site (Jan *et al.* 2011). The resulting insertion was 1876 base pairs. The progeny were screened for a green fluorescent pharynx. All PCR amplicons are listed in Table 3. PCR amplicons are 3.1 (*P_myo-3_*), and 3.2 (*GFP*) and were injected at 0.136 – 0.434 pmol/µl. B) The size of the insert was increased to 2669 bp by including the *unc-54* 3′ untranslated region (UTR, yellow). PCR amplified amplicons are 3.1 (*P_myo-3_*), 3.3 (*GFP*), and 3.4 (*3′ UTR)* and were injected at 0.213 – 0.371 pmol/µl. C) The overall size of the insert was increased to 3557 bp with the addition of a fourth fragment encoding *mCherry* (red). In all panels, the resulting progeny were screened for a red or green fluorescent pharynx. PCR amplicons were injected at 194 - 0.443 pmol/µl and are 3.1 (*P_myo-3_*), 3.5 (*GFP*), 3.6 (*mCherry*), and 3.7 (*3′ UTR*). The number of successfully injected P0 broods scored was 16, 34, and 21 for A, B, and C, respectively. Total F1 *dpy-10* edits (non-fluorescent plus fluorescent) screened was 339, 547, and 324 for A, B, and C, respectively. Data are the percent integrations among the fluorescent *dpy-10* edits. The number of *dpy-10* positive worms screened is in parentheses.

We next sought to determine the size limitations of mmCRISPi. Using crRNA2 with the *Mos1* transposon on chromosome I (*ttTi4348*), we tested the integration of fragments ranging in size from 1,800 bp to 3,500 bp. The reporter consisted of two to four overlapping PCR fragments: the *myo-2* promoter to direct pharyngeal expression, the coding sequence of GFP or mCherry for visualization, and the *unc-54* 3′ UTR (Figure 3). Interestingly, the largest insert size (∼3,500 bp) and highest fragment number (4) tested yielded the largest percent of integrations (Figure 3C). Overall, the insertion sizes tested demonstrated that large integrations are achievable by mmCRISPi.

**Figure 4:**
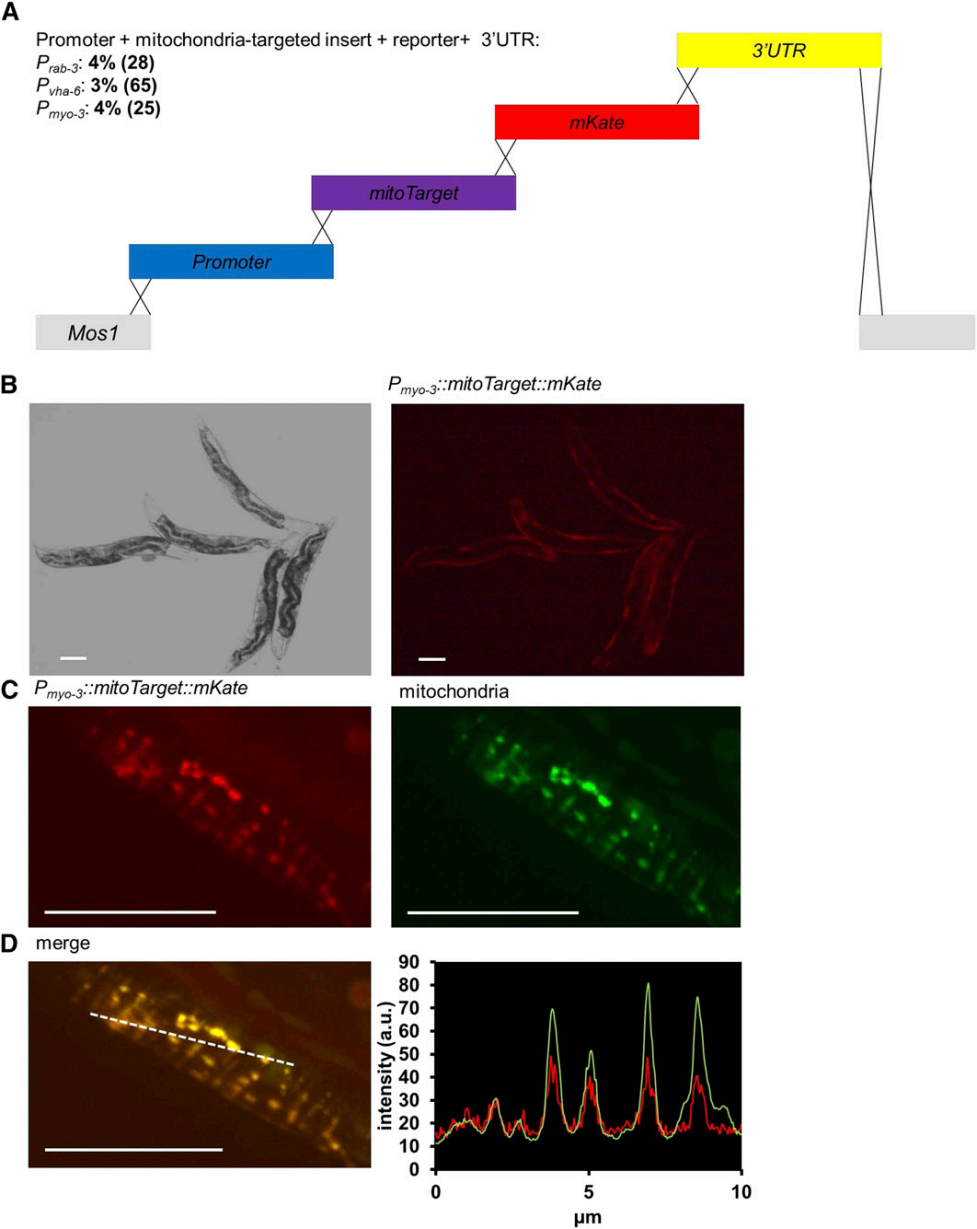
Single copy expression of an integrated mitochondria-targeted transgene. A) Schematic (not to scale) of the insertion shows the double strand break in the *Mos1* transposon (gray) using crRNA2. The break was repaired using four amplicons, which included a promoter (blue), a mitochondria-targeted (SDHC target sequence) transgene (purple), the fluorescent protein mKate (red), and 3′ UTR of *unc-54* (yellow). The *rab-3*, *vha-6*, and *myo-3* promoters drove expression in neurons, intestines and body wall muscles cells, respectively. The total insert size was 3955, 3505 and 5113 base pairs for *rab-3*, *vha-6*, and *myo-3* promoter constructs, respectively. All homology regions overlap by 35 bases, as indicated by crossed lines. The resulting progeny were screened for a red fluorescent mitochondrial pattern in the promoter specific tissue. Data are the percent integrations among the fluorescent *dpy-10* edits. The number of *dpy-10* positive worms screened is in parentheses. All PCR amplicons are listed in Table 3. PCR amplicons 4.1 (*P_rab-3_*), 4.2 (*mitoTarget*), 4.3 (*mKate*), and 4.4 (*3′ UTR*) were injected at 0.202 - 0.213 pmol/µl in mixes for *P_rab-3_*. PCR amplicons 4.5 (*P_vha-6_*), 4.2 (*mitoTarget*), 4.3 (*mKate*), and 4.4 (*3′ UTR*), were injected at 0.256 - 0.417 pmol/µl for mixes containing *P_vha-6_*. PCR amplicons 4.6 (*P_myo-3_*), 4.2 (*mitoTarget*), 4.3 (*mKate*), and 4.4 (*3′ UTR*) were injected at 0.170 – 0.171 pmol/µl in mixes containing *P_myo-3_*. The number of successfully injected P0 broods scored was 9, 15, and 14 for *P_rab-3_*, *P_vha-6_*, and *P_myo-3_*, respectively. Total F1 *dpy-10* edits (non-fluorescent plus fluorescent) screened was 165, 325, and 124 for *P_rab-3_*, *P_vha-6_*, and *P_myo-3_*, respectively. B) Bright field and red fluorescence image of an animal expressing the red fluorescent, mitochondria targeted transgene under a body wall muscle cell promoter (*P_myo-3_*::*mitoTarget*::*mKate*::*unc-54 3′ UTR*). Scale bars 100 μm. C) Confocal images showing red fluorescent transgene and MitoTracker Green FM stained mitochondria in a single body wall muscle cell. Scale bars 10 μm. D) Merged confocal images showing mitochondria-targeted red fluorescent transgene signal overlaps with MitoTracker Green FM stained mitochondria. Scale bar 10 μm. Profile plot of red and green intensities shown generated from the dotted white line in the merged image, indicating correct mitochondrial targeting. Intensity shown in arbitrary units (a.u.).

As a proof-of-principle, we then applied mmCRISPi to recombineer an exogenous, subcellular-targeted transgene. We fused a mitochondria targeting sequence from the rat complex II subunit *Sdhc* of the electron transport chain to *mKate2* and drove expression via several different tissue-specific promoters (Figure 4A). The overall insert size ranged from ∼3,500 to 5,100 bp depending on which promoter was used. Despite the range in sizes we found frequency of integration was consistent across gene promoters and sizes (Figure 4A). Tissue specific expression was observed by red fluorescence (Figure 4B). Further, correct mitochondria targeting was confirmed by overlapping mKate fluorescence with a green fluorescent mitochondrial dye, MitoTracker Green FM (Figure 4C & 4D). These results demonstrate the ability of mmCRISPi to express novel, single-copy integrated genes with correct subcellular targeting.

## Discussion

The mmCRISPi approach uses toolkits readily available and well-characterized (Paix *et al.* 2015; Frøkjær-Jensen *et al.* 2012; Paix *et al.* 2016; Arribere *et al.* 2014; Frøkjær-Jensen *et al.* 2008). The versatility of mmCRISPi allows the user to choose the site of integration and engineer a transgene seamlessly without cloning. The most common mosSCI insertion strains have been validated and target transcriptionally active regions. Importantly, mmCRISPi allows users to alter the integration site by changing the injection strain without altering the composition of their injection mixes.

Numerous *Mos1* insertion alleles are available (Vallin *et al.* 2012). Among the *Mos1* alleles characterized for mosSCI, we tested three different integration sites to offer alternative chromosome options to facilitate genetic crosses. We found that the insertion site on chromosome II (*ttTi5605*) yielded more integration events. While this strain may be more conducive to mmCRISPi, the close proximity of the *dpy-10* co-CRISPR edit may interfere with outcrossing. The chromosome II site may be compatible with other co-CRISPR approaches, such as *pha-1* co-conversion (Ward 2015), or alternatively if fluorescence readout is possible, the efficiency is high enough that transgenesis can potentially be verified in the absence of a co-CRISPR marker.

In addition to choosing the site of integration, mmCRISPi allows a user to customize the transgene using PCR amplicons. Some genes can be difficult to clone and time consuming. Recombineering transgenes *in vivo* through HDR uses PCR fragments with short, ∼35 base sequences with terminal homology (Paix *et al.* 2015; Kemp *et al.* 2007). mmCRISPi is an à la cart transgene integration method, and altering the tissue or the subcellular localization can be achieved by changing a single fragment. Previous reports demonstrated that multiple fragments, as opposed to a single large fragment, were more efficient (Paix *et al.* 2016). Building upon these findings, we demonstrated large insertions using multiple fragments are possible.

The *in vivo* construction of transgenes can generate arrays that are maintained extrachromosomally (Kemp *et al.* 2007). Although we injected promoter-less fluorescent proteins, we found expected fluorescent patterns in worms lacking an integration event, suggesting the formation of an extrachromosomal array. The inclusion of an inducible negative selection marker (Frøkjær-Jensen *et al.* 2012) could limit false positives from extrachromosomal arrays. However, extrachromosomal arrays and integrated transgenes are not mutually exclusive, thus the negative selection would also decrease the number of potential integrations. Future approaches to streamline the selection of integration events could use entry strains. New methods, such as Single-copy Knock-In Loci for Defined Gene Expression (SKI LODGE) developed strains containing a promoter and 3′UTR separated by a crRNA target sequence (Silva-Garcia *et al.* 2019). Since a reporter gene is not injected with a promoter, integration is required for expression. However, current tools are restrictive since the number of promoters and chromosomal position of the insert are limited.

mmCRISPi generated large genome insertions, and new developments in CRISPR/Cas9 technology could increase the frequency and efficiency. For example, optimized Cas9 concentrations and hybrid donor repair templates can greatly improve edit frequency (Dokshin *et al.* 2018). In addition, optimized variants of the Cas9 enzyme could be used to increase efficiency and decrease off-target effects (Zetsche *et al.* 2015; Chatterjee *et al.* 2018). Overall, mmCRISPi combines the advantages of tools freely available to the *C. elegans* community to efficiently express user defined genes in a timely manner.
